# Identification of Clinically Relevant Subgroups of Chronic Lymphocytic Leukemia Through Discovery of Abnormal Molecular Pathways

**DOI:** 10.3389/fgene.2021.627964

**Published:** 2021-06-28

**Authors:** Petr Taus, Sarka Pospisilova, Karla Plevova

**Affiliations:** ^1^Central European Institute of Technology, Masaryk University, Brno, Czechia; ^2^Department of Internal Medicine – Hematology and Oncology, University Hospital Brno, Brno, Czechia; ^3^Faculty of Medicine, Masaryk University, Brno, Czechia

**Keywords:** chronic lymphocytic leukemia, pathway mutation score, ensemble clustering, extreme gradient boosting, mutation subtypes

## Abstract

Chronic lymphocytic leukemia (CLL) is the most common form of adult leukemia in the Western world with a highly variable clinical course. Its striking genetic heterogeneity is not yet fully understood. Although the CLL genetic landscape has been well-described, patient stratification based on mutation profiles remains elusive mainly due to the heterogeneity of data. Here we attempted to decrease the heterogeneity of somatic mutation data by mapping mutated genes in the respective biological processes. From the sequencing data gathered by the International Cancer Genome Consortium for 506 CLL patients, we generated pathway mutation scores, applied ensemble clustering on them, and extracted abnormal molecular pathways with a machine learning approach. We identified four clusters differing in pathway mutational profiles and time to first treatment. Interestingly, common CLL drivers such as ATM or TP53 were associated with particular subtypes, while others like NOTCH1 or SF3B1 were not. This study provides an important step in understanding mutational patterns in CLL.

## Introduction

Chronic lymphocytic leukemia (CLL) is a genetically and clinically heterogeneous disease. The disease manifestations range from asymptomatic with no need for therapy to an aggressive disease associated with therapeutic resistance and overall survival of less than 3 years (Kipps et al., 2017). CLL is divided into two main diagnostic subgroups based on the somatic hypermutation status of the immunoglobulin heavy chain variable region genes (IGHV; Damle et al., 1999; Hamblin et al., 1999). Clinical heterogeneity within both groups is substantial, nevertheless, patients with unmutated IGHV typically experience a more aggressive disease (Sutton et al., 2017). Over the past decade, genomic studies in CLL have discovered several putative drivers (Landau et al., 2013, 2015; Puente et al., 2015). Mutations in some of the drivers (e.g., mutations in TP53 and ATM genes) are associated with worse clinical outcomes whereas, in other instances, reports of prognostic relevance vary (e.g., NOTCH1 and SF3B1) (Lazarian et al., 2017; Hallek, 2019). Many of the driver genes cluster in specific signaling pathways (Landau et al., 2013, 2015; Puente et al., 2015), however, in a significant proportion of patients, no recurrent mutation has been found (Puente et al., 2015). Still, only a limited set of molecular pathways may be abnormal due to the contribution of non-recurrent mutations that are commonly present, but their impact remains elusive and deserves further elaboration.

Stratification of CLL patients based on the entire mutation profile could improve the accuracy of prognostication as it has been shown in the context of other diagnoses (Papaemmanuil et al., 2016; Schmitz et al., 2018). In acute myeloid leukemia, patients assigned into subgroups based on patterns of co-mutations in 111 driver genes displayed different clinical outcomes (Papaemmanuil et al., 2016). However, this approach is challenging for a disease as genetically heterogenous as CLL. An alternative approach is to use prior knowledge of a protein-protein interaction network to reduce the heterogeneity and classify patients into subtypes (Hofree et al., 2013; Leiserson et al., 2015; Le Morvan et al., 2017). For example, mutations can be aggregated in network neighborhoods using network propagation that spreads the signal from mutated drivers to other functionally related genes in network space (Hofree et al., 2013). A limitation of such approaches, using the protein-protein interaction network, is that the genes involved in a biological process do not always interact physically.

Kuijjer et al. (2018) developed a method for reducing heterogeneity of mutation data using biological pathways. This approach takes into account all genes in a pathway and quantifies the level of disruption of the pathway function. Based on this approach, the authors identified nine pan-cancer mutation subtypes across the 23 cancer types from The Cancer Genome Atlas (Kuijjer et al., 2018). To the best of our knowledge, either network- or pathway-based stratification of CLL patients using mutation data has not been performed until now.

Unsupervised learning, also known as clustering, has been extensively used to gain insight into the underlying structure of complex biological data and has led to discoveries of various cancer molecular subtypes (Noushmehr et al., 2010; Cancer Genome Atlas Research Network, 2011; Hedegaard et al., 2016). However, there are several pitfalls, stemming from the nature of biological data, which must be considered during the clustering analysis to obtain robust and meaningful results (Ronan et al., 2016). These pitfalls may be overcome by the application of a combination of multiple clustering solutions through a consensus approach (i.e., ensemble clustering). In this study, we used sequencing data gathered by the International Cancer Genome Consortium (ICGC) for 506 CLL patients to generate pathway mutation scores and applied ensemble clustering. We extracted abnormal molecular pathways with a machine learning approach and identified groups of CLL patients that differ in pathway mutational profiles, as reflected by the clinical behavior of the disease.

## Results

### Reducing Pathway Signature Redundancy to Enhance Prognostic Subtype Identification

**FIGURE 1:**
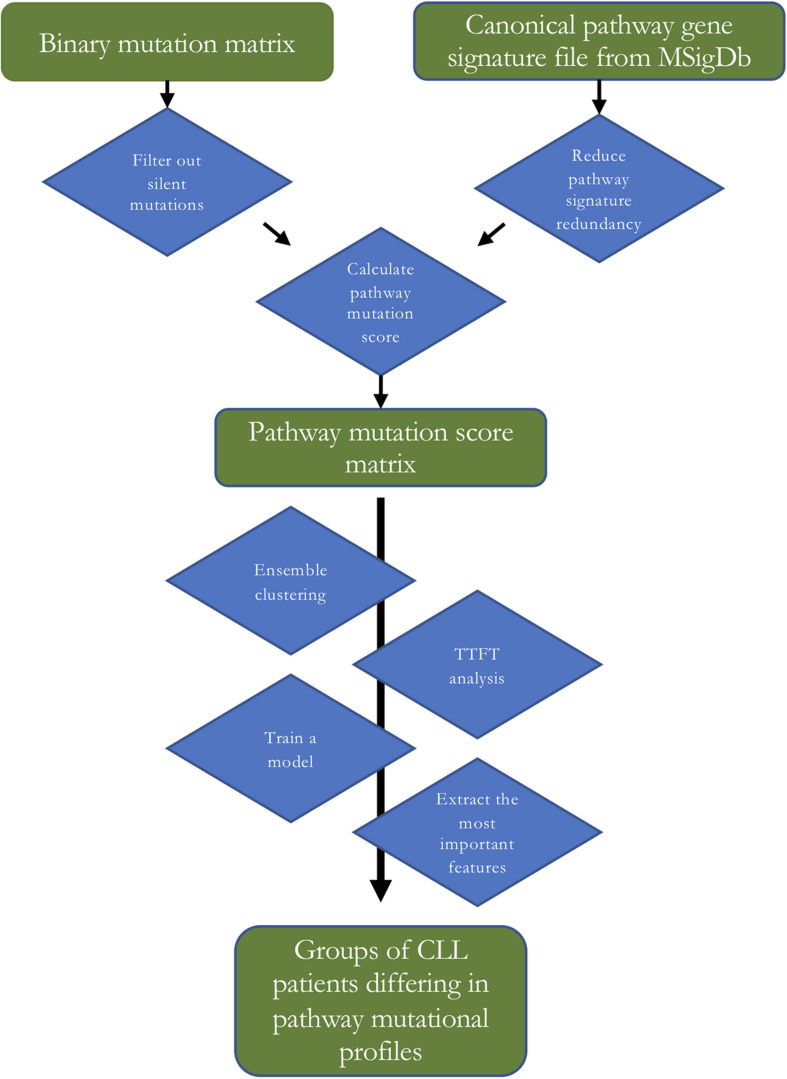
A flow diagram of the analysis. TTFT – time to first treatment.

In the present work, we used 1,329 canonical pathway signatures (covering 8,904 genes) from the collection of curated gene sets (i.e., pathways) from the Molecular signatures database (MSigDB) (Liberzon et al., 2011) gathered from various sources including e.g., BioCarta, KEGG, and Reactome. Combining multiple sources of pathway information often leads to redundancy in the combined dataset that can hinder the downstream analysis. We explored the canonical signature dataset and found out that each gene belonged to 7.6 pathways on average and that the pathway sizes ranged from 6 to 1,028 genes with the median pathway size of 29 genes. This means that most of the pathways contain tens of genes encompassing specific biological processes (see Figure 1 for a flow diagram of the presented analysis).

A set theory algorithms (Stoney et al., 2018) aimed to identify a minimum subset of gene sets required to cover genes in the combined pathway database. We expected that the application of the algorithms would reduce redundancy, decrease dimensionality and lead to the exclusion of large uninformative gene sets. We tested two algorithms, i.e., the hitting set cover and the proportional set cover, that approach pathway reduction in a slightly different way with their unique biases (Stoney et al., 2018). We applied these algorithms with 100 and 99% gene coverage on the canonical signature dataset. Using 99% gene coverage means that we allowed the algorithms not to cover the remaining one percent of genes as the covering of the remaining genes, which tend to have the most overlap with other gene sets, is often at the expense of redundancy reduction. However, this resulted only in marginal improvement of the reduction of redundancy (Table 1), and the excluded genes were mutated in the tested CLL patient samples. In order not to lose this information, for further analyses, we decided to use a reduced pathway dataset with all genes from canonical pathway signatures generated by the hitting set cover algorithm. The hitting set cover algorithm resulted in a 67% reduction of redundancy (from 7.6 to 3.2) and a 58% reduction of dimensionality (from 1,329 to 564) and thus outperformed the proportional set cover algorithm in both the reduction of overall redundancy and decreasing dimensionality (Table 1).

**TABLE 1:** Reducing redundancy using two different set theory algorithms (hitting set cover and proportional set cover) with 100 and 99% gene coverage. The original canonical pathway signatures dataset is described in the first row.

Algorithm	Gene coverage [%]	No. of pathways	Mean pathways per gene	Min pathway size [genes]	Max pathway size [genes]	Median pathway size [genes]
	100	1,329	7.6	6	1,028	29
* Hitting set cover	100	564	3.2	8	389	34
Proportional set cover	100	669	3.5	6	389	30
Hitting set cover	99	513	2.8	8	389	32
Proportional set cover	99	603	2.9	6	389	27

*Star denotes the final solution.*

### Identification of Prognostic Mutation Subtypes Using SAMBAR

In the next step, we tested a method called Subtyping Agglomerated Mutations By Annotation Relations (SAMBAR; Kuijjer et al., 2018), utilizing hierarchical clustering with binomial distance. We applied SAMBAR in default settings, i.e., with subsetting to cancer-associated genes, which resulted in the loss of 22% (*n* = 113) samples without mutation in any of these genes from our patient dataset (*n* = 506). Therefore, we decided not to subset genes in the next analyses. We cut the dendrogram at *k* = 2–7 which means that we grouped the patients into 2–7 groups containing cases with the most similar pathway mutation profiles. We removed clusters of size <20 and tested time to first treatment (TTFT) differences between the subtypes. We identified those solutions with significant differences bearing potential clinical relevance. These concerned *k* = 3 and 5 that, after filtering out clusters of size <20, contained only two clusters (Supplementary Figure 1).

### Identification of Prognostically Relevant Patient Subtypes Using Ensemble Clustering

**FIGURE 2:**
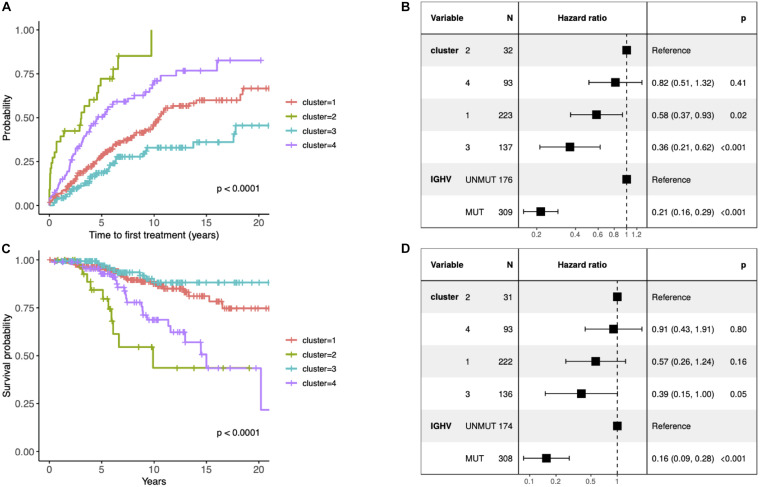
**(A)** Kaplan–Meier curves depicting TTFT for the identified clusters. **(B)** Forest plot of hazard ratios for TTFT according to the identified clusters and IGHV status. **(C)** Kaplan–Meier curves depicting overall survival (OS) for patients within different prognostic subtypes. **(D)** Forest plot of hazard ratios for OS according to the identified clusters and IGHV status. **(B,D)** The column “N” represents the number of samples grouped by the “Variable” column. The column “Hazard ratio” represents confidence intervals for hazard ratios and column “p” *p*-values of test statistics. The present data concern 486 patients with unique information about IGHV status available (i.e., 10 patients without known IGHV SHM status and 1 patient with biclonal rearrangements were excluded). The difference in the total number of patients between panels **(B)** and **(D)** is due to the fact that information about TTFT and OS was available for 485 and 482 patients, respectively.

We further explored whether we could identify subtypes with a greater prognostic value in our cohort that would be defined by distinct pathway mutation profiles. We used a combination of multiple clustering solutions through a consensus approach to cluster pathway mutation scores. We chose distinct clustering algorithms in order to maximize the diversity of the ensemble and therefore to reduce biases due to the selected algorithms (see section “Materials and Methods”). We split data into 2–7 groups and evaluated differences in TTFT for the three best solutions selected based on the proportion of ambiguous clustering (PAC; Şenbabaoğlu et al., 2014). We identified subtypes with significantly different TTFT (log-rank test *p* < 0.05) for clustering solutions splitting data into 5 and 7 groups (Supplementary Figure 2). Clustering samples in 5 and 7 groups produced subtypes of 228, 33, 142, 5, 94 and 141, 57, 93, 47, 41, 66, 57 patients, respectively. As in the previous step, we removed clusters of size <20, therefore, after this filtering step, the clustering solution originally splitting data into 5 groups, contained only 4 groups (Figure 2A).

Since the multiclass classification that we subsequently performed was challenging, we further elaborated the solution with the fewer (i.e., 4) groups in all downstream analyses. First, we evaluated the effect of each subtype characterized by distinct pathway mutation profiles on the TTFT. The subtype with the most favorable prognosis differed from the one with the worst outcome by 20 years in the median TTFT (3 vs 23.4 years) independently of the IGHV status (Figure 2B). We also checked differences in OS, however, they were not independent of the IGHV status in the multivariate analysis (Figures 2C,D).

#### Abnormal Molecular Pathways Extraction

**FIGURE 3:**
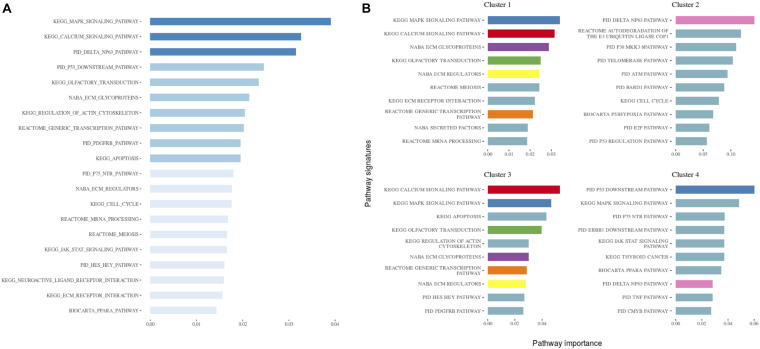
**(A)** The 20 most important pathways for the classification model predicting the identified clusters. There were 84 pathways in the model altogether. Shades of blue represent clustered pathway signatures that have similar importance values. **(B)** The 10 most important pathways characterizing individual clusters of the classification model. Pathway importance was ranked by its information gain, which corresponds to the relative contribution of the pathway to a prediction. The first word in a pathway name denotes a database from which it originates. Pathways shared between clusters are colored.

**FIGURE 4:**
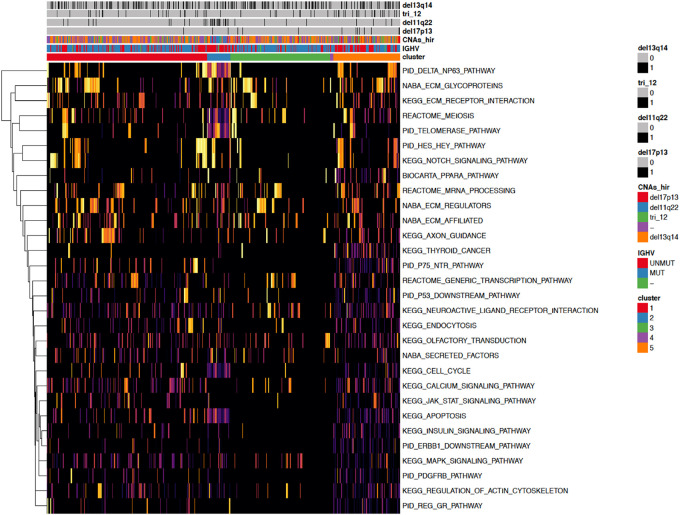
A heatmap showing the 30 most important pathways for the classification model predicting the identified clusters. Copy number alterations are displayed individually and hierarchically clustered according (Döhner et al., 2000).

We next wanted to build a classification model for the identified subtypes, which would be able to assign new cases into existing subtypes. We selected the best model based on a well suited evaluation metric for imbalanced multiclass classification mlogLoss from the five-fold cross-validation, which was 0.54. Next, we evaluated the performance of the final model on a hold-out dataset (*n* = 100), i.e., samples that were not used in any step of the model development, thus representing new, unseen data. The final model used 84 pathway signatures and achieved high prediction performance (0.51 mlogLoss, 0.96 multiclass auROC, and 0.87 multiclass aucPR). The 84 pathway signatures contained 1,504 mutated genes in the dataset. We analyzed protein–protein interactions of mutated genes from each cluster and described gene communities using the fast greedy community detection algorithm. To interpret gene communities, we performed text mining of the column with the description of gene function for each gene and visualized networks (Supplementary Figures 3–6). Then, we extracted the top ten most important features for the model and each subtype separately (Figures 3, 4).

When investigating the most important pathway signatures for each cluster we noticed that the top ten most important pathways in Cluster 2, the cluster with the worst prognosis, all contained the ATM gene. ATM is one of the most commonly mutated genes in CLL (Puente et al., 2015) and the tested cohort, 31 out of 33 patients in Cluster 2 had ATM mutations. This finding prompted us to check the distributions of other common CLL driver genes (Landau et al., 2015; Puente et al., 2015) (i.e., TP53, NOTCH1, SF3B1, MYD88, BIRC3, RPS15, FBXW7 BRAF, EGR2, NFKBIE, XPO1, POT1, ZMYM3, and MGA) in all subtypes (Table 2). We found mutations in TP53 to be solely associated with Cluster 4, containing 94 patients, but no other mutations were specific for a particular subtype.

**TABLE 2:** Distribution of common CLL driver genes among the identified clusters.

Cluster	No. of patients	TP53	ATM	NOTCH 1	SF3B1	MYD88	BIRC3	RPS15	FBXW7	BRAF	EGR2	NFKBIE	XPOI	POT1	ZMYM3	MGA
1	228	0 (0%)	0 (0%)	22 (10%)	19 (8%)	14 (6%)	3 (1%)	3 (1%)	1 (0%)	0 (0%)	5 (2%)	3 (1%)	7 (3%)	6 (3%)	2 (1%)	5 (2%)
2	33	0 (0%)	31 (94%)	6 (18%)	8 (24%)	0 (0%)	1 (3%)	0 (0%)	1 (3%)	1 (3%)	3 (9%)	1 (3%)	0 (0%)	2 (6%)	1 (3%)	2 (6%)
3	142	0 (0%)	0 (0%)	4 (3%)	6 (4%)	0 (0%)	0 (0%)	1 (1%)	0 (0%)	0 (0%)	0 (0%)	0 (0%)	0 (0%)	5 (4%)	2 (1%)	1 (1%)
4	94	15 (16%)	0 (0%)	16 (17%)	8 (9%)	4 (4%)	5 (5%)	0 (0%)	3 (3%)	9 (10%)	1 (1%)	1 (1%)	2 (2%)	4 (4%)	2 (2%)	4 (4%)

#### Identification of Prognostically Relevant Patient Subtypes Within IGHV Subgroups

**FIGURE 5:**
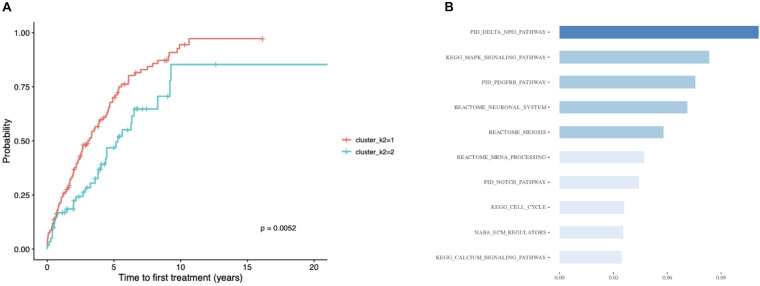
Patients with unmutated IGHV only. **(A)** Kaplan–Meier curves depicting TTFT for patients within different prognostic subtypes. **(B)** The 10 most important pathways characterizing individual clusters of the classification model. Pathway importance was ranked by its information gain, which corresponds to the relative contribution of the feature to a prediction. The first word in a pathway name denotes a database from which a feature originates. Shades of blue represent clustered pathway signatures that have similar importance values.

Considering the substantial impact of IGHV somatic hypermutation status, we then explored whether we could identify subtypes separately within IGHV-mutated vs -unmutated subgroups using the ensemble clustering (Table 3). We found two subtypes among patients with unmutated IGHV differing significantly in median TTFT (3 vs 5.3 years; *p* = 0.0052; Figure 5A), but no separate subtypes among patients with mutated IGHV. The subtype with a more favorable prognosis among IGHV-unmutated cases (median TTFT 5.3 years) consisted of 61 patients, whereas the other one with a worse prognosis (median TTFT 3 years) consisted of 117 patients. Again, we checked the distribution of common CLL driver genes and found mutations in *ATM* and *TP53* only in the cluster with a worse prognosis (Table 4).

**TABLE 3:** Distribution of IGHV somatic hypermutation status among the identified clusters.

Cluster	No. of patients	MUT	UNMUT
1	228	155 (68%)	69 (30.3%)
2	33	4 (12.1%)	28 (84.8%)
3	142	107 (75.4%)	30 (21.1%)
**4**	94	43 (45.7%)	50 (53.2%)

**TABLE 4:** Distribution of common CLL driver genes between the clusters identified within the unmutated IGHV subgroup.

Cluster	N of patients	TP53	ATM	NOTCH 1	SF3B1	MYD88	BIRC3	RPS15	FBXW7	BRAF	EGR2	NFKBIE	XPOI	POT1	2MYM3	MGA
1	117	8 (7%)	26 (22%)	34 (29%)	14 (12%)	0 (0%)	6 (5%)	3 (3%)	4 (3%)	8 (7%)	7 (6%)	3 (3%)	6 (5%)	11 (9%)	4 (3%)	7 (6%)
2	61	0 (0%)	0 (0%)	6 (10%)	9 (15%)	0 (0%)	0 (0%)	1 (2%)	0 (0%)	0 (0%)	0 (0%)	1 (2%)	3 (5%)	5 (8%)	2 (3%)	4 (7%)

Finally, we built a classification model for the identified subtypes and extracted the most important pathway signatures for the model (Figure 5B). The final model used 35 pathway signatures (containing 1,004 mutated genes in the dataset) and achieved good prediction performance (0.92 auROC and 0.85 aucPR).

## Discussion

In the present study, we built a combination of multiple clustering solutions through a consensus approach and applied it to the pathway mutation scores of CLL patients. We identified four clusters differing in pathway mutational profiles and TTFT. Although the identification of prognostic mutation subtypes in the pan-cancer analysis by clustering pathway mutation scores has already been carried out (Kuijjer et al., 2018), to our best knowledge, this is the first attempt to apply a similar approach to a CLL dataset.

We developed machine learning models which classified CLL cases into the identified mutation subtypes with high performance. We leveraged feature importance assigned to pathway signatures by the models to extract subtype-specific pathway mutation profiles. Among the most important pathway signatures, biological processes previously described as recurrently mutated in CLL appeared frequently: namely DNA-damage response, RNA processing, and inflammatory pathways (Hallek, 2019). More importantly, we also identified processes, which have not been described as recurrently mutated in CLL but are known to play a vital role in CLL biology, such as calcium signaling (Lawrence et al., 2013; Martincorena and Campbell, 2015) and pathways involved in cellular motility and interaction (Lazarian et al., 2017). Interestingly, common CLL drivers such as ATM or TP53 were associated with particular subtypes, while others like NOTCH1 or SF3B1 were not (Lazarian et al., 2017). These results suggest that the clinical effect of well-known CLL driver genes depends on mutation background.

We anticipate that the findings of our study will have implications for the improved identification of patients with high-risk CLL, even without well-known CLL drivers. In addition, using pathway mutation scores rather than single-gene approaches could help to identify groups of CLL patients who might respond to specific targeted therapies. This is of importance especially in the light of current treatment options (Hallek, 2019). For example, we hypothesize that patients with affected pathways involved in calcium signaling could respond differently to the treatment with Bruton’s tyrosine kinase inhibitors since calcium signaling can be triggered by BCR pathway stimulation (Chiu and Talhouk, 2018). We believe that our findings will pave the way for the design of new personalized treatment strategies focusing not only on well-known driver genes but also taking into account mutational patterns in particular biological pathways.

## Materials and Methods

### Processing of Somatic Mutation Data

Somatic mutation data were downloaded from a published study (Puente et al., 2015) containing 506 pre-treatment patient samples. Among these, 452 patients were diagnosed with CLL and 54 with MBL. By IGHV somatic hypermutation status, there were 316 IGHV-mutated cases and 179 IGHV-unmutated cases, 1 biclonal, and 10 undetermined cases. Silent mutations were filtered out and only mutations in protein-coding regions and splice sites were kept. Then mutational matrix was binarized. The average number of affected genes per patient was 14.1. If not stated otherwise all analyses were performed using R software v3.4.4 (R Core Team, 2020). The Supplementary Figures 3–6 were prepared using R software v3.4.4 (R Core Team, 2020) and Cytoscape software v3.7.1 (Shannon et al., 2003).

### Reducing Pathway Signature Redundancy

Proportional and hitting set cover algorithms (Stoney et al., 2018) were applied on the canonical pathway gene signature file “c2.cp.v6.2.symbols.gmt” downloaded from MSigDb (Liberzon et al., 2011). The gene coverage threshold was set to 100 and 99%, meaning that one percent of the genes from the original dataset would be missing in the resulting reduced datasets. Then, the excluded genes were checked, whether they were mutated in the patient cohort, and properties of the pathway sets (such as median pathway size, mean paths per gene, min/max pathway size, and the number of pathways) were calculated and compared before and after reduction. Based on this evaluation, a pathway signature dataset was created by the application of a hitting set cover algorithm with a 100% gene coverage threshold was chosen for further analysis.

### Mutation Subtype Identification Using SAMBAR R Package

The *sambar* function from the SAMBAR package v0.2 was used to identify CLL mutation subtypes. The function subsets somatic mutation data to 2,352 cancer-associated genes, divides the number of mutations by the gene length, and calculates gene mutation score. Then, it corrects for sample-specific mutation rate and for the number of pathways each gene belongs to, and de-sparsifies gene mutation score into pathway mutation score when it corrects for pathway length. In the final step, it performs hierarchical clustering with binomial distance on the pathway mutation score.

However, gene length normalization is only a partial correction for the background mutation rate, which depends on other features including 3D structure, gene expression level, and GC content (Martincorena and Campbell, 2015). Additionally, we hypothesized that gene length normalization is relevant in tumor types with a high mutation rate but in tumors with low mutation rates, including CLL (Lawrence et al., 2013), this correction could introduce noise in the data. Therefore, we decided to omit this correction and binarized the mutation score. The function was further modified to exclude subsetting to cancer-associated genes. Then, it was applied on the whole patient cohort following the instruction on https://github.com/mararie/SAMBAR and in Kuijjer et al. (2018) with the reduced pathway signature file as a signature input for the *sambar* function. Two to seven subtypes were assessed.

### Identification of CLL Subtypes Using Ensemble Clustering

The pathway mutation score was calculated using the *sambar* function but without gene length correction and subsetting to cancer-associated genes. De-sparsification of somatic mutation data resulted in a data matrix containing 503 patients and 553 pathway signatures. The pathway signatures that were affected in less than 10 patients were removed, leaving us with 502 patients and 344 pathways. Ensemble clustering was applied on pathway mutation score for the whole cohort and the cohorts with mutated and unmutated IGHV using the R package diceR v0.5.2 (Chiu and Talhouk, 2018). Four distance-based and two non-distance-based methods were included. The distance-based methods were the following: Ward linkage hierarchical clustering, divisive analysis clustering, partition around medoids, and k-means. As the distance metrics for these algorithms, binomial and Mahalanobis distance and random forests proximity converted to distance were used. The non-distance-based methods were the following: spectral clustering using radial-basis kernel function and self-organizing map with hierarchical clustering. Ninety percent (90%) resampling on five replicates was performed and the 2–7 subtypes were evaluated. The average PAC across the clustering results was assessed and half of the solutions with the lowest PAC were selected for further evaluation. Subsequently, the K-modes algorithm was applied to combine the results of the clustering.

#### Associations With Clinical Parameters

Publicly available clinical data were downloaded from the ICGC Data Portal and information about TTFT as an important clinical parameter was extracted. A log-rank test was used to identify whether the found subtypes differed in TTFT (*p*-value < 0.05). All the P values were adjusted for multiple comparisons using the Benjamini–Hochberg correction. If more solutions differed in TTFT statistically significantly, the one with the least subtypes was chosen for further analysis. A Multivariate Cox regression model was fitted to assess the independent prognostic impact of IGHV somatic hypermutation status of each subtype in the outcome of the patients.

### A Classification Model for the Identified Subtypes

The Extreme gradient boosting algorithm (Chen and Guestrin, 2016) is a machine learning approach that combines a large number of weak learners (i.e., slightly better than random guessing) based on decision trees into a single strong learner (i.e., a prediction model). The prediction model can then be applied to a single sample to calculate a group probability. Here we aimed to build a classification model for the identified subtypes and to extract the most important features for each cluster in the prediction model. The extreme gradient boosting algorithm from R package xgboost v0.82.1 was implemented using pathway mutation scores as the input features. Before a model tuning, highly correlated features (*r* > 0.7/*r* < −0.7) and clusters smaller than 10 patients were removed leaving us with 497 patients and 317 pathway signatures. Then, data were split randomly into a training set (80% of patients) and a test set (20% of patients). To find the best number of rounds for the algorithm, it was run with subsample parameter set to 0.25 and the following parameter settings of learning rate and depth of trees were tested: 0.01, 0.05, 0.1, 0.3, and 4, 6, 9, respectively. The algorithm was stopped after 100 rounds without improvement of multiclass Logarithmic Loss function (mlogloss), which was evaluated using a five-fold CV. The algorithm was run again with an optimized number of rounds and selected parameter setting, which minimized mlogloss. Feature importance was ranked by its information gain, which corresponded to the relative contribution of the feature to a prediction. The process of the parameter tuning was repeated with half of the most important features and then in the following repetitions with 3/4 of the most important features until mlogloss started increasing. The performance of the model with optimized parameters and extracted features was tested using mlogloss, multiclass auROC, and multiclass aucPR. An information gain of the features was extracted for each subtype separately.

## Data Availability Statement

The original contributions presented in the study are included in the article/Supplementary Material, further inquiries can be directed to the corresponding authors.

## Ethics Statement

Ethical review and approval was not required for the study due to the secondary use of published data. The original written informed consents with the research use of the data were collected by the ICGC consortium.

## Author Contributions

PT designed the research and performed the analysis. PT and KP analyzed the results and wrote the manuscript. KP and SP supervised the study and critically evaluated the manuscript. All authors contributed to the article and approved the submitted version.

## Conflict of Interest

The authors declare that the research was conducted in the absence of any commercial or financial relationships that could be construed as a potential conflict of interest.
