# Supplementary material for: Identification of Clinically Relevant Subgroups of Chronic Lymphocytic Leukemia Through Discovery of Abnormal Molecular Pathways
Source: Front Genet. 2021 Jun 28;12:627964. doi: 10.3389/fgene.2021.627964 (PMC8273263; doi:10.3389/fgene.2021.627964)
Supplement: Supplementary file 1 [file Data_Sheet_1.PDF]

Identification of clinically relevant subgroups of chronic lymphocytic leukemia through discovery of abnormal molecular pathways

Authors

Petr Taus[1], Karla Plevova[1,2], Sarka Pospisilova[1,2]

[1] Central European Institute of Technology, Masaryk University, Brno, Czech Republic
[2] Department of Internal Medicine – Hematology and Oncology, University Hospital Brno and Faculty of Medicine, Masaryk University, Brno, Czech Republic

Supplementary Material

LEGENDS

Supplementary Figure S1. Kaplan-Meier plots depicting TTFT for the clusters identified using SAMBAR that performs hierarchical clustering with binomial distance without gene length correction and subsetting to cancer-associated genes. We cut the dendrogram at k=3 (A) and k=5 (B) and removed clusters of size < 20 patients. In case of k=3 (A) or k=5 (B), the blue curve represents the cluster of n=26 and n=23, respectively, whereas the red curve represents n=476 and n=458 cases, respectively.

Supplementary Figure S2. Kaplan-Meier plot depicting TTFT for identified clusters using ensemble clustering with k=7.

Supplementary Figure S3. Network representation of affected genes from the 84 extracted pathway signatures in the cluster 1. Node color represents the results of fast greedy community detection algorithm. Node size is based on log normalized betweenness centrality. Edges represent protein-protein interactions. Wordclouds represent the most frequent words from the description of function for each gene in the respective community. Number denotes respective community – 1: green, 2: blue, 3: pink, 4: dark green and 5: red.

Supplementary Figure S4. Network representation of affected genes from the 84 extracted pathway signatures in the cluster 2. Node color represents the results of the fast greedy community detection algorithm. A node size is based on log normalized betweenness centrality. The edges represent protein-protein interactions.

Supplementary Figure S5. Network representation of affected genes from the 84 extracted pathway signatures in the cluster 3. Node color represents the results of fast greedy community detection algorithm. Node size is based on log normalized betweenness centrality. Edges represent protein-protein interactions. Wordclouds represent the most frequent words from the description of function for each gene in the respective community. Number denotes respective community – 1: orange, 2: pink, 3: dark green, 4: green and 5: blue.

Supplementary Figure S6. Network representation of affected genes from the 84 extracted pathway signatures in the cluster 4. Node color represents the results of fast greedy community detection algorithm. Node size is based on log normalized betweenness centrality. Edges represent protein-protein interactions. Wordclouds represent the most frequent words from the description of function for each gene in the respective community. Number denotes respective community – 1: dark green, 2: green and 3: blue.

FIGURES

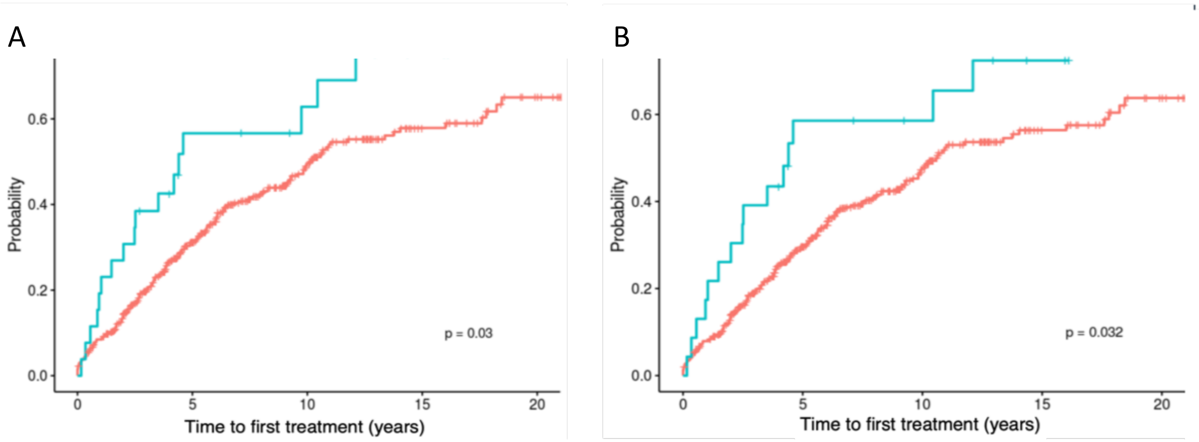

Supplementary Figure S1

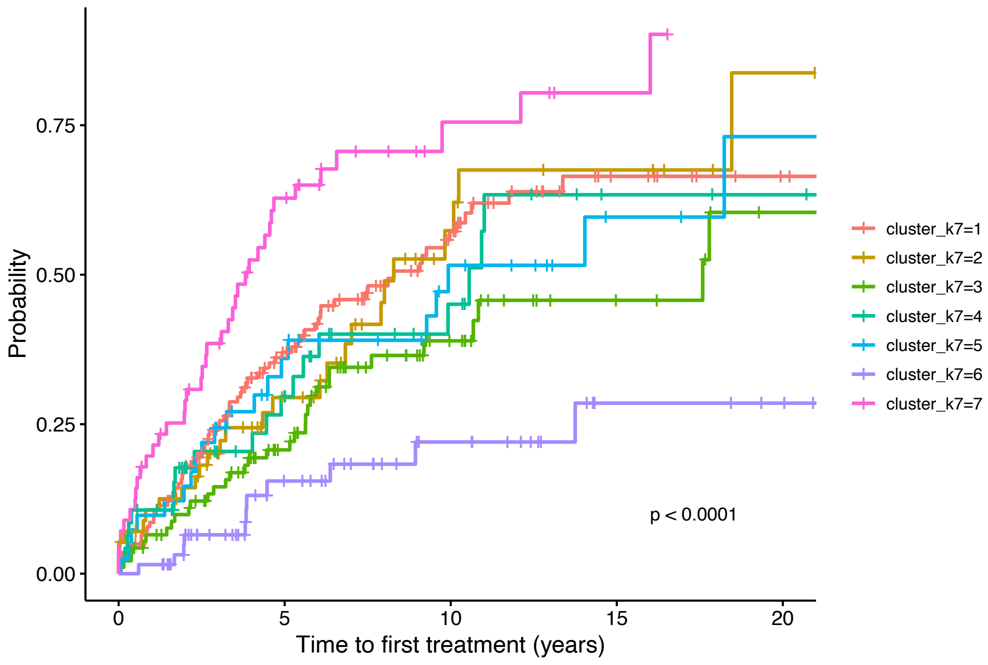

Supplementary Figure S2

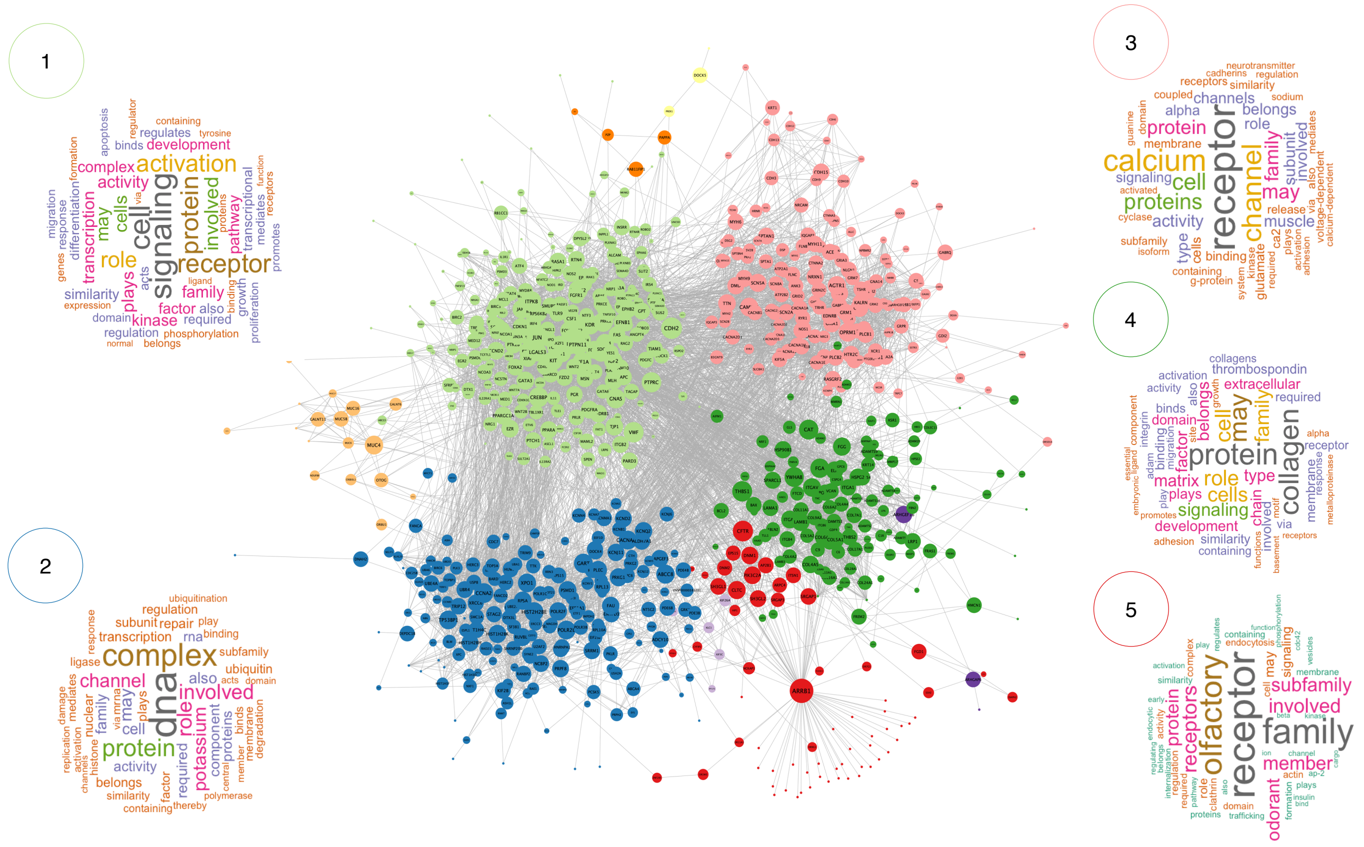

Supplementary Figure S3

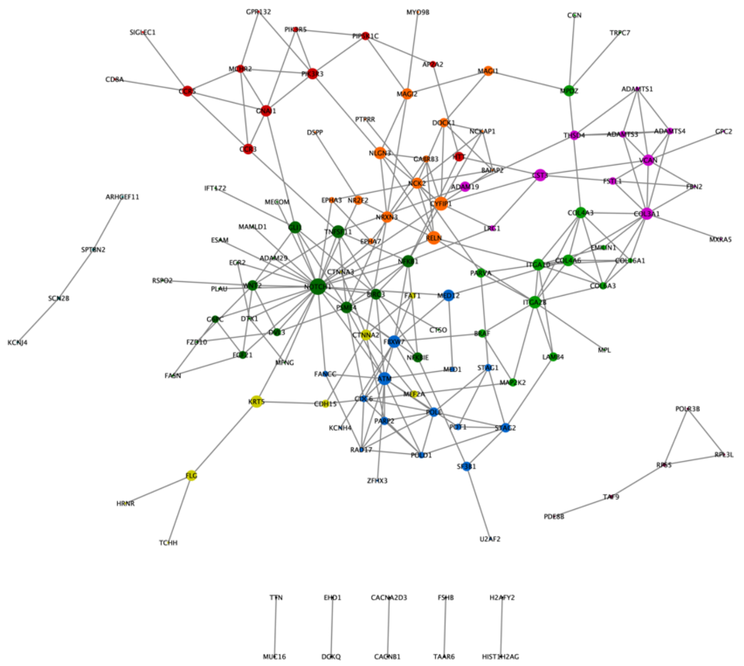

Supplementary Figure S4

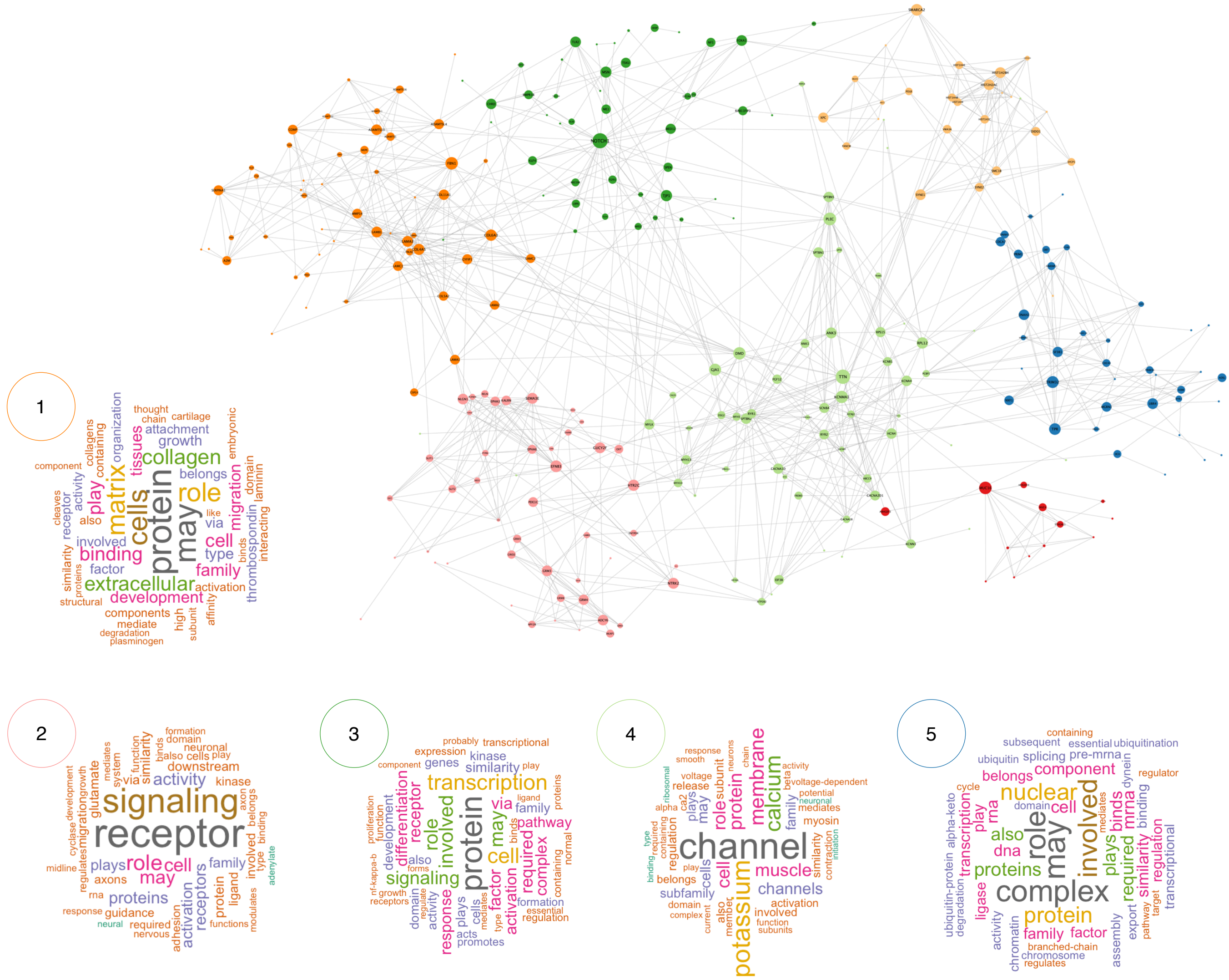

Supplementary Figure S5

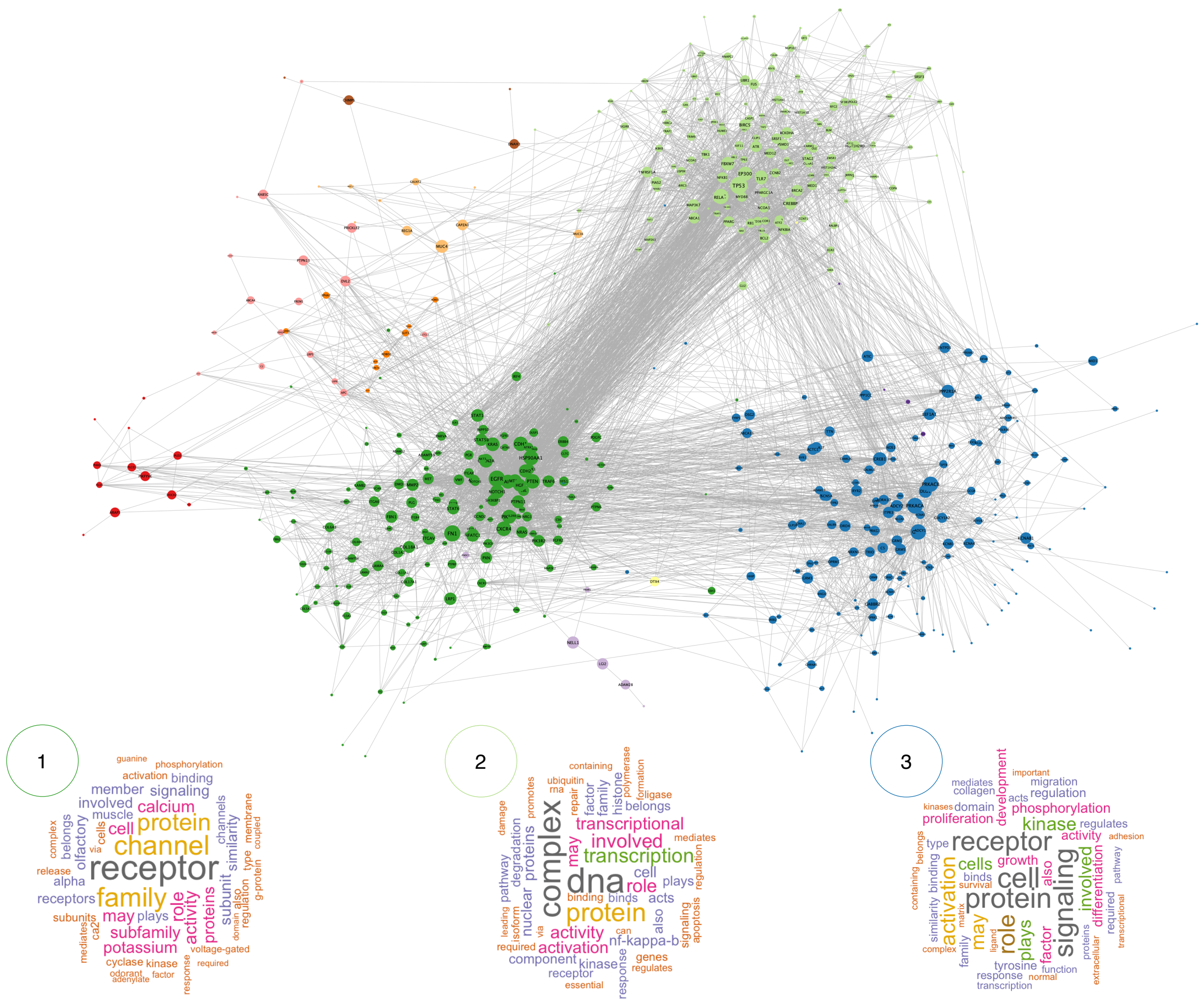

Supplementary Figure S6
